# Combination effect of Pentoxifylline and L-carnitine on idiopathic oligoasthenoteratozoospermia

**Published:** 2014-12

**Authors:** Najme Moslemi Mehni, Ali Asghar Ketabchi, Ebrahim Hosseini

**Affiliations:** 1 *Islamic Azad University, Science and Research Branch, Fars Province, Shiraz, Iran.*; 2 *Physiology Research Center, Kerman University of Medical Sciences, Kerman, Iran.*

**Keywords:** *Idiopathic*, *Oligoasthenoteratozoospermia*, *Male infertility*, *Pentoxifylline*, *L-carnitine*

## Abstract

**Background:**

Pentoxifylline (PX) is a methyl xanthine derivative that influences the sperm motion characteristics and L-carnitine (L-C) is an amino acid that is naturally produced in the body. In general, separate administration of PX and L-C has been reported to be effective on preserving sperm motility in vitro, and also when is consumed orally by the Idiopathic oligoasthenoteratozoospermia (IOAT) patients.

**Objective:**

The aim of this study was to evaluate any possible effect of a combination of L-C and PX on sperm characteristics and improving the type of assisted reproductive techniques (ART) in a group of patients with unexplained oligoasthenoteratozoospermia.

**Materials and Methods:**

Two hundred twelve infertile men with IOAT in a double-blind, placebo-controlled, randomized clinical trial were allocated for this study. They randomized to four groups. Group I received PX/ and L-C (each one, twice daily), group II, PX and placebo, group III, L-C with the placebo, and group IV, received placebo tablets. Finally, we compared pre and post intervention sperm parameters and ART procedures between groups.

**Results:**

While the use of PX and L-C are only improved sperm motility, but their combined uses improved all sperm parameters, especially the sperm count. Also the combination of PX and L-C was effective on improving the ART procedures (p<0.01).

**Conclusion:**

Our results demonstrate that the combination use of PX and L-C is useful in improving of sperm parameters in IOAT patients and also, improve ART procedures in this group of patients.

## Introduction

Idiopathic Oligoasthenoteratozoospermia (IOAT) is related to defective spermatogenesis and is a condition of abnormally low sperm count, motility, and high count of dysmorphism spermatozoa in the ejaculate, the origin of which is unknown and often regarded as undetectable by common laboratory methods. Approximately 30% of OAT patients are diagnosed as idiopathic and usually the most severe OAT are caused by idiopathic testicular abnormality (1). Although some known diseases such as a varicocele, cryptorchidism and hypogonadism are definable causes for OAT and infertility, but over 25% these cases have not known cause for their abnormal semen analysis (2, 3). 

There are very few valid and scientific study has been conducted in the treatment of patients with idiopathic oligoasthenoteratospermia and their infertility, and currently in this field only traditional and empirical medicine or compounds with unknown effects are used (4). However, most of IOAT patients for the conception of their wives will be referred to ART procedures, and the main determinant factor in the ART type choice is quality of sperm motility. IOAT can be classified as: isolated astheno ±teratospermia with normal sperm count, moderate (sperm count <20×10^6^/mL and >5×10^6^/mL); and or severe (sperm count <5×10^6^/mL) (5). 

The total number of motile sperm after sperm preparation has been shown to predict the outcome after IVF and a count of 500,000 has been suggested as a cutoff value for IVF, although others recommend a number of >10^6^ progressively motile sperm (6). So even when there is the final ART procedures choice, improving sperm quality causes the better results and better type of ART (7, 8). For improving of the quality of sperm parameters, at least for final ART decide, plenty of medical therapy was recommended for IOATS, as Antioxidant, Hormone Pharmacotherapy, Nonsteroidal Anti-Inflammatory Drugs, Pentoxifylline (PX), carnitine, Vitamin C and E, Selenium, N-Acetyl Cysteine, Zinc and Folic Acid, Mast Cell Stabilizers, Lycopene, and Adrenergic Antagonists (9).

According to results of recent studies on the effect of administration of PX and L-carnitine (L-C) on sperm parameters in OAT patients, in a new experience we have assessed combination effect of PX and carnitine in this regard. PX is a competitive nonselective phospho-diesterase inhibitor that raises intracellular cAMP and reduces inflammation by inhibiting TNF-a and leukotriene synthesis, and also it has been reported to decrease (reactive oxygen species )ROS production, and to preserve sperm motility in vitro and improve semen parameters in vivo (10-13). 

The study of Tesarik *et al* showed that in unselected asthenospermic patients, PX improves sperm motion characteristics such as curvilinear velocity, path velocity and beat cross frequency, but without modification of motile spermatozoa percentage (14). In other studies PX in selected asthenospermic patients with detectable steady state levels of ROS improved sperm motion parameters, the presence of ROS in semen has detrimental effect on sperm motion parameters and in these conditions high dosage orally administered PX as antioxidant and ROS scavenger was shown to increase sperm motility (14, 15).

L-C is an amino acid; approximately 25% is synthesized from lysine and methionine and naturally is produced in the body and is a water-soluble antioxidant that mostly derived from the human diet. Carnitine at extracellular and intracellular levels may play a significant role in sperm energy metabolism, and provide the primary fuel for sperm motility during epididymal passage (13). The healthy epididymes contains Carnitines in both free and acetylated forms for use of spermatozoa via mitochondrial β-oxidation of long chain fatty acids, as a main transferring system of the acyl to the mitochondrial CoA, and by decreasing fatty acid oxidation restore the phospholipid composition of mitochondrial membranes (16-21). 

According to The results of previous studies the oral carnitine components have a favorable effect on sperm motility of men with IOAT. Some authors suggest a daily carnitine dose of 3 gr that is given for about four months has significant improvement of sperm motility from pretreatment levels, but some believe even a higher dose of 4 gr per day over shorter treatment duration (two months) also increases significantly progressive sperm motility (22-23). A more recent, controlled study in which 2 gr carnitine was administered daily and they showed the most significant improvement in motility was seen in the groups with lower baseline motility (18, 24). The IOAT, particularly severe cases are the major causes of male factor infertility, which finally may influence the pregnancy success rates following ART (25). 

Therefore, a potential pitfall exists for these infertile men where their infertility problem resides in multiple sperm parameters abnormalities, though the sperm motility plays major role in IOAT treatment, and there are some evidences that the improvement of sperm motility with application of only PX or LC may not only be beneficial for improving these complex IOAT abnormalities, so in this study we have tried to investigate the simultaneous effect of PX and LC with known and different effects on complex semen parameter abnormalities of IOAT patients, and any benefits on ART procedures of these patients.

## Materials and methods

In a double-blind, randomized clinical trial from May 2008 to August 2012, from 255 infertile men with IOAT who had been referred serially to Bahonar Hospital in Kerman, Iran, 212 patients (25-40 years old) as our sample size by informed written consent was allocated to this study.The study was approved by Ethics Committee of Kerman University of Medical Sciences, and supported financially by physiology center of Kerman University of Medical Sciences. 

The diagnosis was made after the medical assessment, which included a thorough and comprehensive history and physical examination with emphasis on the evaluation of the male reproductive system (i.e., varicocele, testicular volume evaluation) and genital ultra-sonography and body mass indexes (BMI) assessment. Two semen samples from these men were evaluated one before and another after intervention for basic parameters (semen samples preparation and analysis was performed according to WHO 2010 recommendations) and presence of antisperm antibodies,and also by commercial radioimmunoassay kit we evaluated hormonal profiles (serum FSH, LH, testosterone, estradiol, and prolactin ) of them. 

so included men were infertile with IOAT, with healthy and fertile wives. The exclusion criteria in this study were existence of genital abnormalities (undescended testes, varicocele, atrophy of testes), occupational chemical exposure history, systemic diseases, and abnormal semen volume, pH, agglutination or viscosity, serum hormonal abnormalities (FSH, LH, testosterone, estradiol, and prolactin) and also who had wives with known fertility risk factors (confirmed by gynecologist). 

Finally included patients randomized by Bloch method to four groups. Group I received 400 mg PX and 500 mg L-C twice daily, group II, 400 mg PX and placebo twice daily, group III, 500 mg L-C with placebo twice daily and group IV, placebo with placebo tablets twice daily for duration of three months. Sperm concentration, total and fast progressive motility (%) and morphology (%) were analyzed for each sample. As the choice of ARTs treatments (IUI, IVF, and ICSI) in male factor infertility is often based on semen quality (proportion of motile spermatozoa), so categorized choice of ART procedures in this study was, ≥5 million/ml sperm for IUI,1-5 million/ml sperm for IVF, and ≤1 million/ml sperm for ICSI. 

**Statistical analysis**

All data in this study analyzed by SPSS software (Statistical Package for the Social Sciences, version 19.0, SPSS Inc., Chicago, Illinois, USA)., our main analysis was based on estimation of ANOVA Repeated measures, and also we used One way ANOVA, Paired t test and Crosstab from 2010 Excel software and statistical significance was defined when p<0.05.

## Results

The demographic findings of all patients in four groups were matched, and they had (6.2-8.9 years) infertility duration ranges, with 73% primary and 27% secondary infertility (p=0.788), and 54.28% of the patients had opium addiction without significant difference between groups (p=0.454). The history of surgery for their infertility was %48.9 and this was the same between them (p=0.96) (Figure 1). By linear regression analyses the relation of the above mentioned variables to sperm parameters was assessed (Table I-II, Figure 1). After intervention 23 patients excluded from study (3 patients for drug intolerance in groupI, and 20 patients for uncooperative in group II and III). 

The results of interventions in this study showed that the single use of PX and L-C only improve sperm motility rate in IOAT patients, but the combined use of them cause improving all main sperm parameters (Table III). Finally the comparison of sperm parameters before and after intervention for ART type showed, the simultaneous use of PX and L-C improved the selection of ARTs type in our patients, so after intervention the ARTs type, from group 1-4, the IUI selection were 28.3%, 8.2%, 12%, and 0%, respectively, and IVF selection were 28.3%, 30.6%, 23.5%, and 5% respectively, also mean and standard deviation of IUI and IVF after intervention in group I were 6.25±6.3 and 11.25±5.7 respectively (p<0.05) (Figure II).

**Table I:** Demographic characteristics of patients (group I-IV)

**Characteristics**		**Group I**	**Group II**	**Group III**	**Group IV**
Number*		53	49	51	59
Age (year)**		32 ± 2.3	28 ± 3.6	30 ± 1.7	30 ± 4.6
Addiction opium and or cigarette*		25	28	22	30
BMI**		19±2.8	20±4.5	25 ± 1.2	22 ± 2.2
Type of infertility:					
	P (primery)*	37	30	35	42
	S (secondary)*	16	19	16	17
Infertility duration (years)**		5.5 ± 3.2	7.5 ± 1.2	4.5 ± 3.8	8.5 ± 1.5

* : values are presented as numbers.

** : values are presented as mean±SD.

**Table II:** Semen parameters after intervention

**Groups**	**Count (×10** ^6^ ** )**	**Motility (%)**	**Morphology (%)**
I	20 ± 4.5	35 ± 2.2	17.6 ± 3.2
II	8 ± 6.5	22.5 ± 5.2	4 ± 3.7
III	9.3 ± 1.7	24.6 ± 1.5	2.2 ± 2.2
IV	0.8 ± 1.8	3.3 ± 2.7	1.7 ± 0.8

All values are presented as mean±SD.

**Table III:** Comparison of semen parameter chenges in four groups (after vs. before treatment)

**Semen parameters**	**Group I (PX+LC)**	**Group II (PX)**	**Group III (LC)**	**Group IV (Placebo)**
Count	0.001	0.062	0.044	0.565
Motility	0.045	0.046	0.005	0.112
Morphology	0.052	0.064	0.065	0.086

Data presented the p-values. Paired t test and Crosstab and Statistical significance was p<0.05.

**Figure I:**
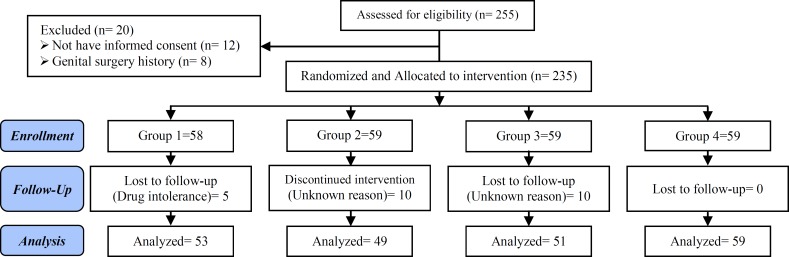
Consort flow diagram (study selection strategy).

**Figure II:**
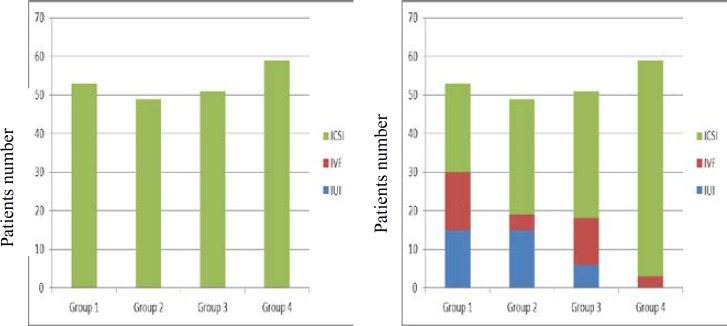
Comparison of ART procedures before (left: only ICSI indicated) and after (right: by improved Semen qualities, IVF and IUI indicated) intervention.

## Discussion

Approximately 30% of OAT cases are considered idiopathic, meaning that no cause can be found with common clinical, instrumental or laboratory methods. Treating IOAT can be hard, although a variety of drugs and dietary supplements are available, many are prescribed without any rationale and without any evidence supporting their efficacy (23). Usually there are two alternative approaches to treatment of IOAT: pharmacological therapy and/or ART. The purpose of pharmacological therapy is to stimulate spermatogenesis, and to increase sperm quality, maturity and the ability. In this study we have investigated the effect of PX and L-C as pharmacotherapy on improving semen parameters of patients and ARTs procedure choices. Both PX and L-C are from the nutraceutics pharmacy group which derives from the combination of the words ‘nutrition’ and ‘pharmaceutics’. They have been used in various clinical trials, either singly or rare in combination by other agents, to improve sperm parameters especially sperm motility (24). 

Regarding sperm motility, there was a highly significant increase in sperm progressive motility in our treated groups. This is in agreement with other studies that revealed a significant improvement in sperm motility, in our study this result is partially related to hyper activation and the acrosome reaction following activation by PX, and also due to L-C effect (25). PX has been demonstrated to increase testicular sperm motility when it added to culture media (26). It inhibits the breakdown of cAMP and it is known that intracellular cAMP concentration plays a central role in cell energy which in turn sustains sperm motility and finally the anti-oxidant activities of PX have demonstrated in infertile men with IOAT (27). The increase of cAMP leads to increase progressive sperm motility. The cAMP plays an important role in the glycolytic pathway of the sperm and, through can affect glycolysis. It can influence the energy generation required for sperm motion (28). 

Also the beneficial effects of L-C on the quality of sperm and/or spontaneous pregnancy outcome in patients with idiopathic asthenozoospermia have been demonstrated in a number of clinical studies (29-31). High concentrations of carnitine, a 3-hydroxy-4-trimethylaminobutyric acid, are present in both seminal plasma and spermatozoa. Carnitine plays a major role in the transport of fatty acids through mitochondrial membranes and in the intracellular storage of acetate moieties derived from acetyl-co (32). L-C and acetylcarnitine are important for sperm metabolism and providing energy for use by spermatozoa (33, 34). However, it has been shown that the seminal plasma of oligoasthenozoospermic men contain lower levels of L-C compared with fertile men (35). Consequently, these observations create a rationale for treatment with L-C especially with PX in IOAT patients.

ART procedures will be the final therapeutic attempts in most of IOAT patients. Although ARTs have been proposed as a possible solution for IOAT men, but they are expensive, not universally available, and have limited success, and also the success rate of ARTs relates strongly to quality of semen parameters and so the better types of IOAT will lead to select the less-invasive reproductive techniques and also with better outcomes. Therefore, it is necessary to improve semen parameters before to decide to ART procedures. As our research has showed the main effect of PX and L-C singly or combined can improve sperm motility, and in fact sperm motility factor is base of OAT intensity classification and guide for choice of ART procedures. Sperm motility even has an extremely important role for positive results in even in IUI procedures and, and even more important is the ability to maintain motility, quality over time as insemination and ovulation may not coincide exactly (36). 

IOAT can be classified as: isolated astheno± teratospermia (no alteration in semen concentration or ≥20×10^6^/mL); moderate (sperm concentration <20×10^6^/mL and >5×10^6^/mL); or severe (sperm concentration <5×10^6^/mL), and the improvement of sperm motility was more marked in those patients who presented a lower concentration of normo-kinetic spermatozoa before therapy (number of spermatozoa with total motility <10×10^6^ and forward motility <5×10^6^/ ejaculate) (37, 38). However, there is today a variety of medical treatments for improving sperm parameters of IOAT patients, and in this study, we have tried to assess clinically the combination effects of PX and L-C on infertile IOAT patients, although there is a paucity of research in the literatures about in vitro use of combined PX and L-C in semen of infertile men with IOAT, and also we have determinated the effects of these drugs on ARTs types between them if finally they need these techniques. 

The results of simultaneous use of PX and L-C in this study may be due to synergistic or additive effect of each drug, and or other different may involve in semen parameters improving by them as qualifying of sperm motility by an increase in LDH-C4 enzyme activity (39). In current study besides assessing the combined effects of PX and L-C on main sperm parameters, also we tried to determine the relationship between body mass index (BMI), duration of infertility, surgery history, addiction and types of infertility variables, with sperm parameter status in IOAT patients. But except infertility type (primary vs. secondary) and smoking addiction (cigarette, opium), other above mentioned variables have not statistically relation with semen parameters of our IOAT patients. 

With strong evidence in literatures the smoking adversely affects male and female fertility and smokers are more likely to be infertile, also we have not found significant relationship between other data as BMI, Although subjects with both extremes of BMI have poor spermograms (40). However we think any known risk factor for sperm parameters abnormalities should be assisted and corrected beside main therapies.

## Conclusion

In conclusion, this study has demonstrated a clear positive effect of the oral administration of combined PX and L-C on the all sperm parameters especially kinetics of the spermatozoon in these subjects affected with OAT. 

Thus, we propose this type of therapeutic approach as a possible technique for treating selected forms of male infertility, especially in view of the serious lack of certain treatments that are efficacious on the mechanisms of activation and maintenance of sperm parameters. Also by improving ART types it is indicated for those that are preparing male partner in couples for intrauterine insemination programs or other ARTs.

## Conflict of interest

There was no conflict to be stated.

## Note

***Registration ID in IRCT: IRCT201310131981N3***

***This article extracted from M.Sc. thesis. (Najme Moslemi Mehni)***
